# Task and Resting-State Functional Connectivity Predict Driving Violations

**DOI:** 10.3390/brainsci13091236

**Published:** 2023-08-24

**Authors:** Uijong Ju

**Affiliations:** Department of Information Display, Kyung Hee University, Seoul 02447, Republic of Korea; juuijong@khu.ac.kr; Tel.: +82-02-961-0858

**Keywords:** functional connectivity, resting state, functional connectome, aberrant driving, violations, error, lapses

## Abstract

Aberrant driving behaviors cause accidents; however, there is a lack of understanding of the neural mechanisms underlying these behaviors. To address this issue, a task and resting-state functional connectivity was used to predict aberrant driving behavior and associated personality traits. The study included 29 right-handed participants with driving licenses issued for more than 1 year. During the functional magnetic resonance imaging experiment, participants first recorded their resting state and then watched a driving video while continuously rating the risk and speed on each block. Functional connectome-based predictive modeling was employed for whole brain tasks and resting-state functional connectivity to predict driving behavior (violation, error, and lapses), sensation-seeking, and impulsivity. Resting state and task-based functional connectivity were found to significantly predict driving violations, with resting state significantly predicting lapses and task-based functional connectivity showing a tendency to predict errors. Conversely, neither impulsivity nor sensation-seeking was associated with functional connectivity. The results suggest a significant association between aberrant driving behavior, but a nonsignificant association between impulsivity and sensation-seeking, and task-based or resting state functional connectivity. This could provide a deeper understanding of the neural processing underlying reckless driving that may ultimately be used to prevent accidents.

## 1. Introduction

Recent neuroimaging studies have leveraged functional connectivity (FC; the functionally integrated relationships between different brain regions) to predict various personal features. Resting-state FC (rsFC) and task-based FC (tbFC) have been employed for this purpose, successfully predicting various personal features such as attentional processing [1,2,3], creative ability [4], personality [5,6] and performance on perceptual and cognitive tasks [7,8,9]. Despite these advancements, a gap in FC research remains, as relatively few studies have sought to apply these predictive capabilities to personal features with direct implications for real-world behaviors such as risky driving, which is closely associated with the occurrence of fatal vehicular accidents [10].

In the context of functional magnetic resonance imaging (fMRI), several studies have investigated the neural networks underlying risky driving behaviors. For example, one study compared drivers with and without frequent traffic offenses and found differences in the activity of the superior temporal gyrus [11]. Additionally, research on risk-taking during a computerized driving test revealed that the striatum and medial prefrontal cortex are more specifically associated with risk-taking behavior [12]. Another study suggested that urgent driving situations, which necessitate cognitive control for safe behavior, induce increased frontal activation [13]. Overall, various brain networks have previously been demonstrated to be associated with aberrant or risky driving behaviors.

Since various networks are associated with aberrant driving behavior, understanding the neural mechanisms underlying this behavior may be enhanced by studying FC, which refers to the level of covariation between spatially distributed brain networks [14]. In this regard, several previous studies have investigated associations between FC and drivers’ cognitive states, such as tbFC with distraction [15], and both tbFC and rsFC with driving fatigue [16,17] and sleepiness [16], which are indirectly associated with aberrant driving behavior. Consistent with the aforementioned studies, aberrant driving behavior may be associated with rsFC, as individual intrinsic factors or personality traits are associated with rsFC [18,19] and may induce aggressive driving behavior [20]. Additionally, aberrant driving behavior could be associated with tbFC, since situational factors can play a role in inducing drivers’ violation intentions [21], which may be associated with driving situations associated with tbFC. However, compared with other cognitive states such as distraction by secondary tasks [15], fatigue from driving for long periods [17], or external manipulation of participants’ conditions [16], it is relatively difficult to intentionally induce aberrant driving behavior since it is difficult to manipulate emergency situations as drivers may exhibit varied behaviors in dangerous driving scenarios, even under the same situations [22]. Thus, to address this issue, I used recorded driving videos and asked participants to continuously evaluate perceived risks and driving speeds since the subjective evaluation of current driving situations can elicit different emotions that may in turn induce aberrant driving behavior [23]. The advantage and innovation of using this method, as opposed to conventional driving simulations, lie in its relative ease of providing consistent experimental conditions to induce tbFC associated with aberrant driving behavior. Given these unique characteristics, I hypothesized that rsFC and tbFC could predict aberrant driving behavior based on FC recorded during rest and while watching a driving video. To the best of my knowledge, this is the first study to investigate the association between aberrant driving behavior and both rsFC and tbFC.

In the present study, the driving behavior questionnaire (DBQ) widely used to measure aberrant driving behavior, including violations, errors, and lapses, was employed [24].

The term “violations” refers to intentional actions that directly violate traffic rules, such as going over the posted speed limit or running a red light. Errors are actions that are still thought to increase a driver’s risk of an accident while not directly violating traffic laws. Errors originate from problems with information processing, whereas violations heavily rely on motivation [25]. Contrastingly, ‘lapses’ are characterized as insignificant mistakes that are not thought to have contributed to a crash [26].

Additionally, since aberrant driving behavior is associated with personality traits [27], I investigated the personality traits sensation-seeking and impulsivity, which were shown to be associated with aberrant driving behavior [28,29] and accidents [30,31]. I also hypothesized that sensation-seeking and impulsivity could be predicted from rsFC and tbFC.

Based on this framework, I evaluated whether connectivity-based analyses could predict such driving behaviors and personality traits and reveal the associated brain networks by comparing the tbFC and rsFC results. The previously published paradigm for predicting behavior from FC [32] has some crucial advantages that make it well-suited to providing valuable data for this study, as this paradigm can reveal FC between pre-defined brain networks [33].

In summary, I hypothesized that it is possible to decode aspects of driving behavior and driving behavior-related personality traits from FC.

## 2. Materials and Methods

### 2.1. Experimental Design

**Figure 1:**
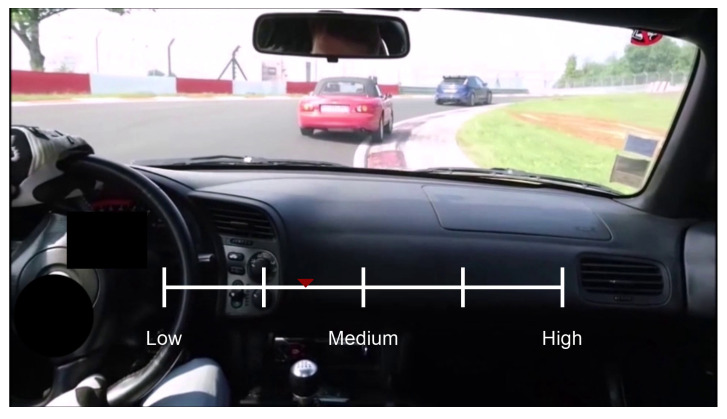
Screenshot of the experiment. Risk and speed are rated by sliding a slider using a trackball and the current rating is indicated by the red arrow. Positions 1, 3, and 5 on a Likert scale are labeled “Low”, “Medium”, and “High”, respectively.

In this study, four driving videos from YouTube were used with the same racing track and similar perspectives (video 1: https://youtu.be/7BwDs3ja-WA; video 2: https://youtu.be/F3IHb8RFV_g; video 3: https://youtu.be/mj7dkeQcNPM; video 4: https://youtu.be/QYV3yWshHLI; accessed on 10 July 2023). I edited the video from the starting point of the racing track to restrict each video to 2 min in length. Subsequently, I used an fMRI-compatible trackball (Current Designs, Philadelphia, PA, United States) to implement continuous rating inside the scanner that was required to rate perceived risk in the first block and speed in the second block (as shown in Figure 1). Additionally, the presented order was counterbalanced across participants to minimize the order effect, and after the experiment, the participants were asked to complete the driving behavior, sensation-seeking, and impulsivity questionnaires from the outside scanner.

### 2.2. Participants

In total, 29 right-handed participants were recruited (21 men; mean age = 22.2 years; standard deviation (SD) = 2.86). The majority of the participants were men as in South Korea, men were more likely to use cars and roads [34]. All participants had owned a driver’s license for >1 year at the time of the experiment (mean = 3.2 years; SD = 2.43). None of the participants had a history of a neurophysiological illness or another ailment that would have prohibited them from participating and none of the participants were involved in serious accidents prior to participation in the study. All participants provided informed consent after the experimental protocol was explained to them and before they undertook the fMRI scanning procedure. This study was approved by the local Ethics Committee of Korea University (KUIRB-2020-0207-02).

### 2.3. Driving Behavior and Personality Measures

I used a 27-item version of the DBQ, as reported in the original paper [35], which included driving violations, errors, and lapses. I also included sensation-seeking [36] and impulsivity [37] as additional items; these were translated and validated to the Korean version of impulsivity (Cronbach’s alpha = 0.8) [38] and sensation-seeking scales (Cronbach’s alpha = 0.76–0.83) [39].

### 2.4. Procedure

**Figure 2:**
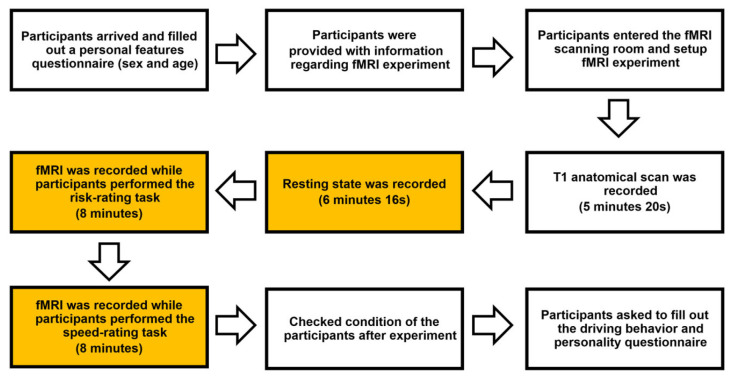
Overall experimental procedure. Yellow sections indicate the procedure used to construct rsFC and tbFC.

Prior to participation, the participants were provided with information on the fMRI experiment. They entered the scanning room after receiving all explanations and instructions, and extra care was taken to ensure that they had a trackball in their right hand before being scanned. First, a T1 anatomical scan was recorded, followed by a resting state scan for 6 min and 16 s. Subsequently, the researcher was alerted that the experiment had begun. In the first block, four 2-min videos were shown to participants in a counterbalanced order, and participants used a trackball to continually score their perceptions of risk while watching the videos. In the second block, the same four videos were presented in a counterbalanced order and participants continuously rated their speed perception. A total of 8 min video presentations made up one block, and 16 min of fMRI recording was used to build the tbFC for the analysis. After the experiment, the researcher checked the condition of the participant and asked them to complete the driving behavior and impulsivity and sensation-seeking questionnaires (see Figure 2 for the overall experimental procedure).

### 2.5. fMRI Data Acquisition

To acquire the MRI data, I used a Siemens Magnetom Prisma 3T scanner (Siemens Medical Systems, Erlangen, Germany), which includes a 64-channel neck coil and a SENSE head coil with 20 channels (Center for Neuroscience Imaging Research, Sungkyunkwan University, Suwon, Republic of Korea). A T1-weighted sagittal high-resolution MPRAGE sequence was used to collect structural MRI data (repetition time (TR) = 2300 ms, echo time (TE) = 2.28 ms, flip angle (FA) = 8°, voxel size = 1 × 1 × 1 mm^3^, and 192 axial slices). Whole brain BOLD resting-state fMRI used a gapless, echo-planar-imaging sequence (TR = 2000 ms, TE = 30 ms, FA = 90°, slice thickness = 2.0 mm, voxel size = 2 × 2 × 2 mm^3^, and 188 volumes) and an echo-planar imaging sequence with no gaps was used for functional imaging (TR = 2000 ms, TE = 30 ms, FA = 90°, slice thickness = 2.0 mm, and voxel size = 2 × 2 × 2 mm^3^). Given that the resting state lasted for 6 min and 16 s, the total volume used for rsFC was 188, and since each video lasted for 2 min, the total volume used for tbFC for each block was 240.

### 2.6. Data Preprocessing

The resting state and functional data were pre-processed using default preprocessing in the CONN toolbox (version 21.a, https://www.nitrc.org/projects/conn, accessed on 10 July 2023) [40]. The default preprocessing pipeline included the functional realignment and unwarping that checked excessive head translation or movements and were realigned to the initial volume (none of the participants had head movement > 2 mm), a slice-timing correction that corrected temporal misalignment between different slices, outlier identification that identified potential outlier scans from the global BOLD signal observed that used Artifact Detection Tool (ART) based identification of outlier scans for scrubbing, direct segmentation, and normalization that normalized functional data into MNI space with a 2 × 2 × 2 mm^3^ size using the normalize function, and functional smoothing that used a Gaussian kernel of 8 mm FWHM to smooth the normalized data. All preprocessing was performed using the SPM12 procedure (London, UK; http://www.fil.ion.ucl.ac.uk/spm/; accessed on 1 July 2023) and the default preprocessing value in the Conn toolbox.

### 2.7. Functional Connectivity Construction

The CONN toolbox with a reduced version of the Shen brain atlas [41] (from the original 268 regions of interest, the “unknown network”) was excluded based on network definition from a previous study [33], resulting in 178 regions of interest—(detailed information of 268 regions of interest are in Supplementary Materials Table S1 and Figure S1)—of 2-mm dimensions was used to construct the FC matrix for each participant and each condition. For this, I first extracted BOLD signals from all ROIs during the resting state and each video condition and computed pairwise bivariate correlations of the time series between all ROIs, finally producing a 178 × 178 correlation matrix for the resting state and video conditions. In both rating conditions, four different video conditions existed; therefore, four FC matrices were computed for rate risk and speed. Next, to obtain the average tbFC matrix during the rating of risk and speed, I averaged the FC matrices across risk and speed rating conditions, finally producing a 178 × 178 × 29 matrix for risk and speed rating conditions.

### 2.8. Connectome-Based Prediction

**Figure 3:**
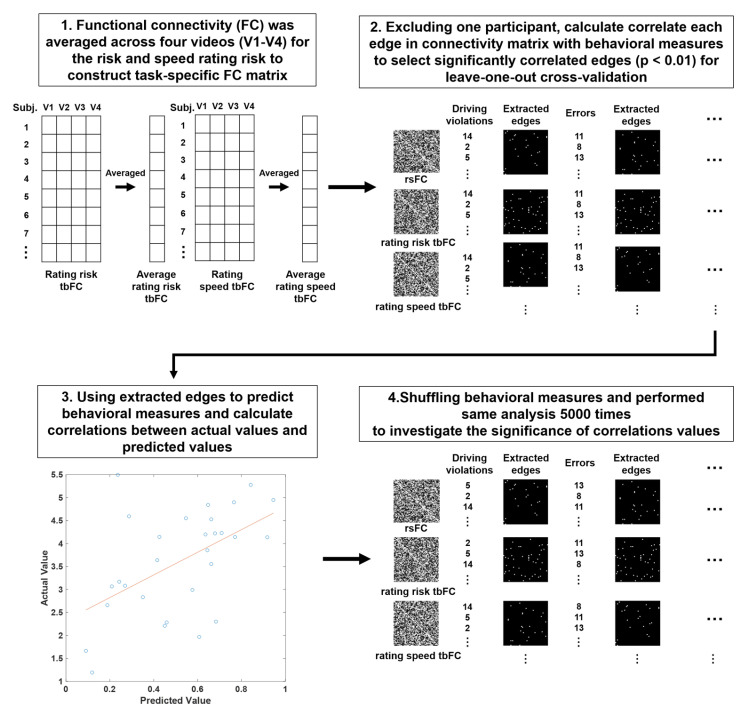
Screenshot of the experiment. Risk and speed are rated by sliding a slider using a trackball, and the current rating is indicated by the red arrow. Positions 1, 3, and 5 on a Likert scale are labeled “Low”, “Medium”, and “High”, respectively.

I referenced the functional connectome-based predictive modeling from a previous study [32] that used FC to predict various behavioral measures. In this study, I used the rsFC and tbFC matrices to predict driving behavior and personality traits. The first analysis setup left one participant out and used the remaining participants to determine a correlation between behavioral measures and the FC matrix that applied a threshold (*p* < 0.01) to extract significant network edges that positively and negatively correlated with behavioral measures. Next, based on the extracted edges, the sum of the connectivity values of all edges that were used to predict the value of leaving out the participant was calculated. This procedure was repeated for all participants, and based on the actual and predicted values, I calculated the correlation and p values of the leave-one-out cross-validation. Next, a permutation test, which shuffled the label of behavioral measures and ran the same analysis 5000 times, was performed. Permutation results were counted as (1 + number of correlation values equal to or greater than the original correlation value)/5001 to investigate the degree of significance of the correlation value (see Figure 3 for the overall procedure). Finally, to visualize network connections that showed a significant contribution based on the extracted edges, I calculated the number of connections between networks/total number of possible connections between the networks to investigate which connections between networks contributed more to predicting driving behavior or personality traits compared with the other connections.

## 3. Results

### 3.1. Behavioral Data Analysis

**Figure 4:**
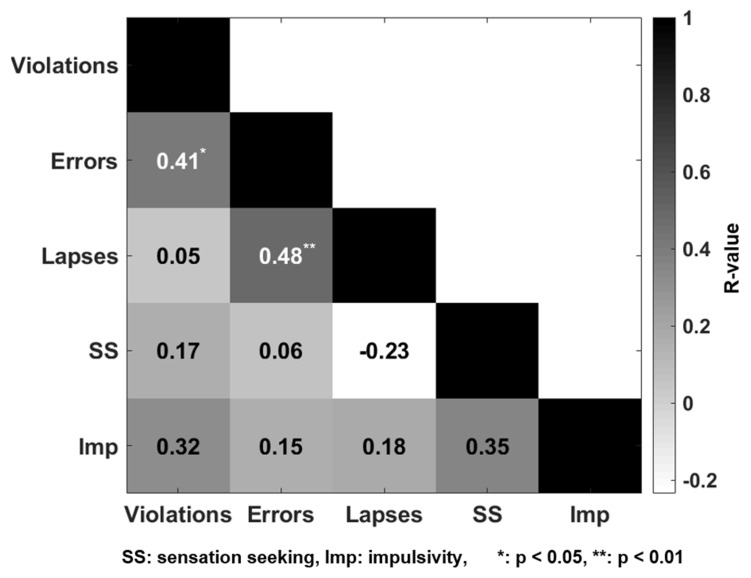
Correlations between the behavioral measure dimensions.

First, inter-correlations between the behavioral data were investigated (as shown in Figure 4). Errors were significantly correlated with violations (r = 0.41; *p* = 0.029) and lapses (r = 0.48; *p* = 0.008). The inter-correlation results confirmed that the behavioral measures were reasonably independent.

### 3.2. Functional Connectivity Predicts Driving Violations

**Figure 5:**
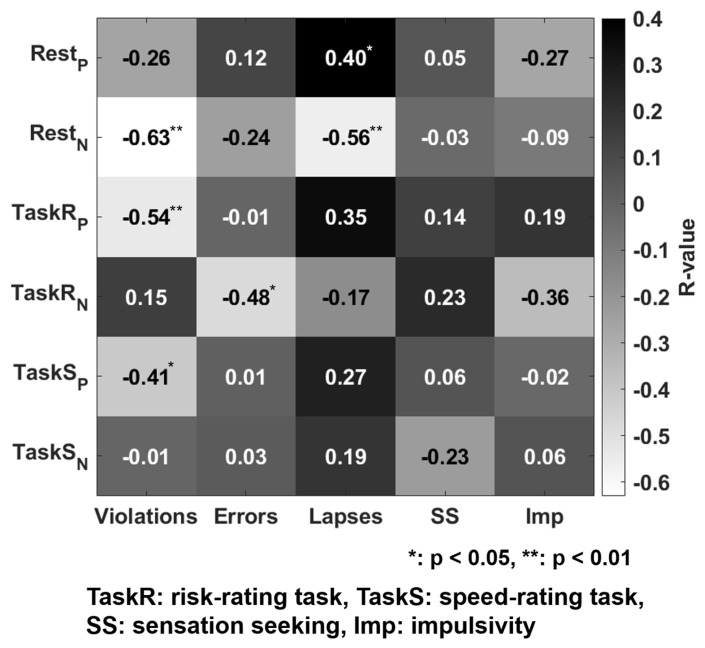
Results of connectome-based predictive modeling for predicting behavioral measures from tasks and resting-state functional connectivity.

Next, based on functional connectome-based modeling, FC was used to predict aberrant driving behavior and associated personality traits (additional information on FC differences between conditions are presented in Figure S2, Supplementary Methods and Results). First, rsFC significantly predicted violations in negative networks (r = −0.63; *p* < 0.001) and lapses in positive and negative networks (positive network: r = 0.40, *p* = 0.033; negative network: r = −0.56, *p* = 0.002). Second, risk-rating tbFC significantly predicted violations in the positive network (r = −0.54; *p* = 0.003) and errors in the negative network (r = −0.48; *p* = 0.009. Finally, speed-rating tbFC only significantly predicted violations in the positive network (r = −0.41; *p* = 0.028). However, none of the FCs significantly predicted impulsivity and sensation-seeking (as shown in Figure 5).

Next, a permutation test was run to evaluate whether the outcomes were significant or the result of random data. All significant results from the above tests were analyzed, and in rsFC, the violation showed significant values (violations: *p* < 0.001; lapses positive: *p* = 0.159; negative: *p* = 0.037). In risk-rating, tbFC only showed significance for violations (violations: *p* = 0.027; errors: *p* = 0.11), while the speed-rating task showed trends for violations (violation: *p* = 0.094). Thus, violations were significantly predicted from rsFC and tbFC, while lapses were predicted from rsFC; however, only risk-rating tbFC showed trends for predicting driving errors.

**Figure 6:**
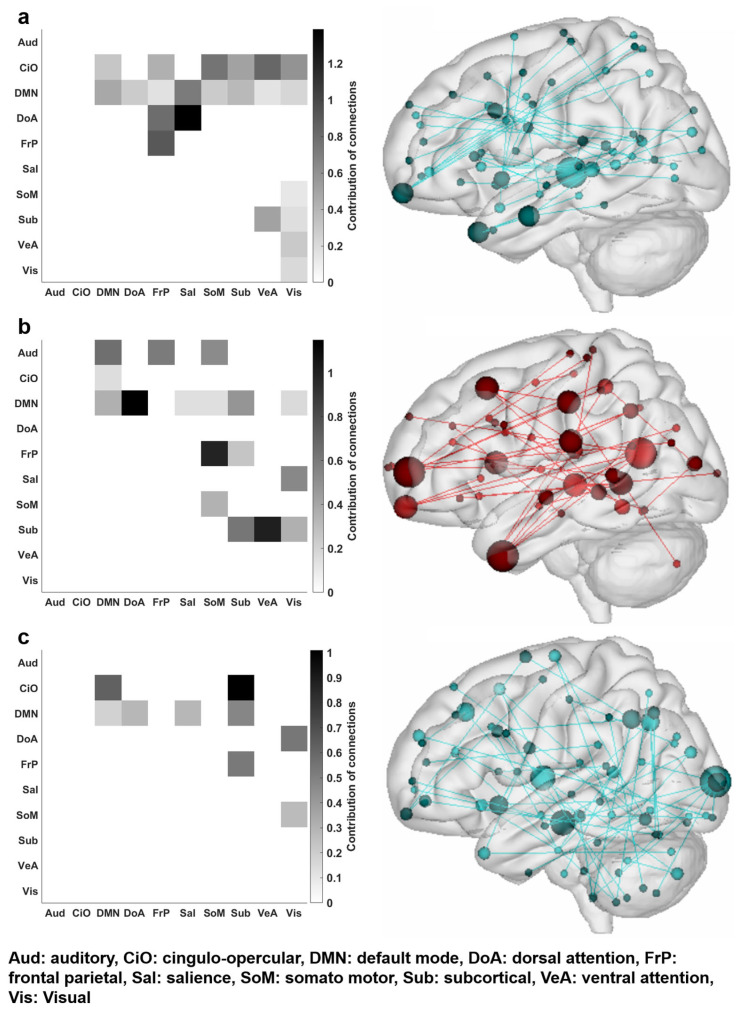
Overall contribution of the connections between brain networks that significantly predict driving behaviors (**a**) resting state—negative network predicted driving violations, (**b**) risk-rating—positive network predicted driving violations, (**c**) resting state—negative network predicted lapses. The color bars represent the connections between the networks (%) and red lines indicated positive networks and blue line indicated negative networks.

Finally, to identify the region that contributes to decoding driving behaviors, the extracted network edges that survived permutation tests were visualized. Both rsFC and risk-rating tbFC to predict driving violations survived the permutation tests. One side of the top contributing network connection was the dorsal attention network with salience networks in rsFC and default mode networks in risk-rating tbFC (as shown in Figure 6a,b, and Table 1). Additionally, subcortical and frontal-parietal networks showed common contributions in both survived network edges, indicating that similar networks contribute to decoding driving violations. Next, rsFC significantly predicted lapses and survived the permutation tests and extraction of network edges showed that the top contributing connections were cingulo-opercular and subcortical networks. Additionally, cingulo-opercular and subcortical networks and the dorsal attention and visual networks were the second and third top contributors, respectively, as shown in Figure 6c and Table 1. Results showed that, compared with driving violations, different network connections predict lapses (additional information on the contributing network that did not survive the permutation test is presented in Supplementary Materials Figures S3–S5).

## 4. Discussion

In this study, rsFC and tbFC were used to predict aberrant driving behaviors and associated personality traits. Results showed that both rsFC and risk-rating tbFC significantly predicted driving violations; rsFC also significantly predicted driving lapses. In addition, risk-rating tbFC partially predicted errors, and speed-rating tbFC partially predicted driving violations; no significant associations were found with impulsivity and sensation-seeking.

In particular, I found that rsFC and risk-rating tbFC significantly predicted driving violations, and the dorsal attention, frontal-parietal, and subcortical networks were revealed as the most common contributing networks. This was consistent with previous studies where gray and white matter in the dorsal attention network were shown to be associated with a high risk of unsafe driving [42,43]. Furthermore, the dorsal attention network has been shown to play a role in the control of stimulus-driven, goal-directed [44], and top-down control of attention [45,46]. This implies that a driving violation habit is associated with attention when a driver intentionally attempts to achieve a goal such as driving fast. Therefore, FC in the dorsal attention network during rest and when performing a driving-related task contributes to predicting driving violations. Additionally, previous studies have shown that the frontal-parietal network is associated with executive control that links to visuospatial processing and logical reasoning [47], which was also shown to be associated with aberrant driving behavior [48]. In this study, the frontal-parietal network or FC between the frontal-parietal and somato-motor network showed a highly contributing network, which implies that executive functions associated with driving violations contribute highly to the frontoparietal network. Finally, subcortical regions also showed high contributions, which may come from the interaction between cortical and subcortical regions that are associated with goal-directed behavior [49]. Since driving violations can occur during reckless driving [50] to achieve a specific goal, FC between subcortical and cortical regions may be associated with driving violations.

Next, I found that rsFC significantly predicted lapses and the top contributing connection was FC between the cingulo-opercular network and both subcortical and default mode networks. A potential reason is that lapses typically denote a temporary decrease in attention or judgment [51] that is consequently associated with cognitive control networks like cingulo-opercular network [52] that are associated with attentional control along with the default mode network [53]. This result is also supported by contributing network connections between dorsal attention and visual networks, which implies that attention-associated visual processing is associated with driving lapses.

Additionally, risk-rating tbFC partially predicted errors, and speed-rating tbFC partially predicted driving violations. A potential reason why risk-rating tbFC only partially predicts errors is that driving errors can be categorized as recognition, decision, performance, and critical non-performance errors [54] and the brain regions associated with understanding and recognizing driving situations such as visual and spatial task-associated areas during the risk-rating task can intensify [55]; therefore, driving error associated with wrong recognition can lead to partial prediction by risk-rating tbFC. Similarly, a potential reason why speed ratings only partially predicted driving violations is that, although speeding is one of the components that constitutes a driving violation [56], the driving conditions on a race track are different from normal driving, and aggressive driving may not appear to be a driving violation to participants and therefore, may not elicit sufficient activation of the brain networks associated with speeding-related violation.

Although previous studies have found that rsFC can predict impulsivity [57] and sensation-seeking [58], no such significant correlations were found in the present study. A potential reason for this is the relatively small sample size compared to those of previous studies [58], or the contrast between whole-brain FC in the present study and region-of-interest-based analyses in previous studies that focused on reward-associated networks [57]. The present study used large-brain networks to make predictions and, therefore, the results may not hold up if only reward-associated brain regions can decode personality traits. Therefore, rsFC may effectively predict sensation-seeking and impulsivity by adopting an ROI-based analysis with a relevant sample size. Additionally, tbFC did not show any significant correlations in the present study. A potential reason is that driving video-based evaluations did not induce brain activity associated with reward-related networks because the continuous evaluation did not receive any positive feedback. Potentially, using a reward-associated behavioral task such as a virtual reality balloon analog risk task [59] may enhance the possibility of predicting impulsivity and sensation-seeking from tbFC.

Finally, despite revealing important insights, this study is not without its limitations. The sample size of this study was restricted in contrast to other rsFC studies [57], potentially limiting the generalizability of the present rsFC results. However, the present study reports both tbFC and rsFC in participants, and sample size limitations of rsFC studies can be mitigated by showing consistent results in tbFC, which has shown a better capacity to elicit significant differences between behavioral phenotypes [1,60,61,62]. Future research should include larger sample sizes and corroborate the prediction accuracy derived from tbFC and rsFC. Additionally, the task undertaken by the participants in the present study does not directly incorporate aberrant behavior videos, which may curtail the overall significance of the findings. Thus, future work would benefit from the inclusion of aberrant driving videos and dynamic assessment within the task to further evaluate the predictability of aberrant behavior from tbFC. Furthermore, although the present study reported top contributing brain networks that significantly predict aberrant driving behavior, other brain networks may also play a role since cognitive processing underlying aberrant driving behavior could require multi-dimensional brain network contributions; therefore, to validate the connectome-based predictive modeling and uncertainty of network contribution in the present work, future studies are also required to validate the contribution of networks in the present study to find common networks that predict aberrant driving behavior.

## 5. Conclusions

The present study investigated the association between FC and aberrant driving behavior, as well as the associated personality traits, using resting state and a driving video assessment task. Overall, the results indicate significant associations between driving violations and lapses, but no significant associations between impulsivity and sensation-seeking, and rsFC and tbFC. Although additional networks that contribute to the decoding of driving behavior exist, the present study suggests that attention-processing-related networks are crucial for decoding aberrant driving behaviors. I expect that this result may serve as a basis for understanding the neural mechanisms underlying aberrant driving behaviors, which could aid in identifying targeted brain regions for cognitive training, specifically in individuals prone to risky driving. Furthermore, the results of the present study showed the possibility that even though the driving video did not directly show aberrant driving behavior, FC from the task was able to predict risky driving habits. This result implies that situations that are difficult to implement directly can be studied by indirect implementation such as recorded videos. In addition, to evaluate the validity of indirect implementation, future studies should also compare stimuli that directly and indirectly show aberrant driving behavior. For example, it would be intriguing to use virtual reality to implement risky driving situations or normal driving with a risk evaluation and compare prediction accuracy between the two methods to further enhance understanding of the neural mechanism underlying aberrant driving behavior. This could lead to improvements in attention, reaction times, and overall driving behavior, ultimately reducing potential accidents.

## Figures and Tables

**Table 1:** Top three contributing connections from surviving edges in the functional connectome-based prediction model. See Figure 6 for FC connections across whole networks.

Condition/Behavioral Measure	Network 1	Network 2	Contribution of Connections (%)
rsFC/driving violations	Dorsal attention	Salience	1.39
	Frontal-parietal	Frontal-parietal	0.91
	Cingulo-opercular	Subcortical	0.79
risk-rating tbFC/driving violations	Default mode	Dorsal attention	1.15
	Subcortical	Ventral attention	1.01
	Frontal-parietal	Somato-motor	0.99
rsFC/lapses	Cingulo-opercular	Subcortical	1.01
	Cingulo-opercular	Default mode	0.63
	Dorsal attention	Visual	0.54

## Data Availability

Data presented in this study are available from the corresponding author on reasonable request due to privacy issues.

## Supplementary Materials

The following supporting information can be downloaded at: https://www.mdpi.com/article/10.3390/brainsci13091236/s1, Table S1: Lists of 268 networks. Unknown networks are excluded from the analysis, Figure S1: Visualization of unknown networks that was excluded in the present study. BioImage Suite Web Connectivity Viewer tool used to categorize unknown networks (BioImage Suite Web; https://bioimagesuiteweb.github.io/webapp/, accessed on 10 July 2023), Figure S2: FC differences between different conditions, (a) FC differences between resting state and risk-rating task, (b) FC differences between resting state and speed-rating task, (c) FC differences between risk-rating task and speed-rating task. The color bars represent the portions of significant FC differences between the conditions (%), Figure S3: Overall contribution of the connections in resting state functional connectivity in predicting behavioral measures, (a) positive network for predicting driving violations, (b) positive network for predicting errors, (c) positive network for predicting lapses, (d) positive network for predicting sensation-seeking, (e) positive network for predicting impulsivity, (f) negative network for predicting errors, (g) negative network for predicting sensation seeking, (h) negative network for predicting impulsivity. The color bars represent the contributed connection between the networks (%), Figure S4: Overall contribution of the connections for task-based functional connectivity when assessing risk to predict behavioral measures. (a) positive network for predicting errors, (b) positive network for predicting lapses, (c) positive network for predicting sensation-seeking, (d) positive network for predicting impulsivity, (e) negative network for predicting driving violations, (f) negative network for predicting errors, (g) negative network for predicting lapses, (h) negative network for predicting sensation-seeking, (i) negative network for predicting impulsivity. The color bars represent the contributed connection between the networks (%), Figure S5: Overall contribution of the connections for task-based functional connectivity when assessing speed to predict behavioral measures. (a) positive network for predicting driving violations, (b) positive network for predicting errors, (c) positive network for predicting lapses, (d) positive network for predicting sensation-seeking, (e) positive network for predicting impulsivity, (f) negative network for predicting driving violations, (g) negative network for predicting errors, (h) negative network for predicting lapses, (i) negative network for predicting sensation-seeking, (j) negative network for predicting impulsivity. The color bars represent the contributed connection between the networks (%).
