# Supplementary material for: Task and Resting-State Functional Connectivity Predict Driving Violations
Source: Brain Sci. 2023 Aug 24;13(9):1236. doi: 10.3390/brainsci13091236 (PMC10526865; doi:10.3390/brainsci13091236)
Supplement: Supplementary file 1 [file brainsci-13-01236-s001.zip › brainsci-2551615-supplementary.pdf]

**Table S1.** Lists of 268 networks. Unknown networks are excluded from the analysis.

| Node | Network | Node | Network | Node | Network | Node | Network | Node | Network | Node | Network |
|---|---|---|---|---|---|---|---|---|---|---|---|
| 1 | Default Mode | 47 | Salience | 93 | Default Mode | 139 | Default Mode | 185 | Default Mode | 231 | Unknown |
| 2 | Unknown | 48 | Default Mode | 94 | Unknown | 140 | Default Mode | 186 | Default Mode | 232 | Unknown |
| 3 | Unknown | 49 | Default Mode | 95 | Default Mode | 141 | Default Mode | 187 | Default Mode | 233 | Default Mode |
| 4 | Unknown | 50 | Dorsal_attention | 96 | Unknown | 142 | Frontal_Parietal | 188 | Default Mode | 234 | Unknown |
| 5 | Default Mode | 51 | Default Mode | 97 | Unknown | 143 | Frontal_Parietal | 189 | Unknown | 235 | Unknown |
| 6 | Default Mode | 52 | Default Mode | 98 | Visual | 144 | Salience | 190 | Default Mode | 236 | Unknown |
| 7 | Frontal_Parietal | 53 | Default Mode | 99 | Unknown | 145 | Default Mode | 191 | Default Mode | 237 | Unknown |
| 8 | Frontal_Parietal | 54 | Ventral_Attention | 100 | Default Mode | 146 | Default Mode | 192 | Ventral_Attention | 238 | Unknown |
| 9 | Salience | 55 | Unknown | 101 | Visual | 147 | Frontal_Parietal | 193 | Unknown | 239 | Unknown |
| 10 | Default Mode | 56 | Unknown | 102 | Unknown | 148 | Default Mode | 194 | Unknown | 240 | Unknown |
| 11 | Salience | 57 | Unknown | 103 | Unknown | 149 | Default Mode | 195 | Unknown | 241 | Unknown |
| 12 | Default Mode | 58 | Unknown | 104 | Unknown | 150 | Salience | 196 | Unknown | 242 | Unknown |
| 13 | Default Mode | 59 | Unknown | 105 | Unknown | 151 | Default Mode | 197 | Default Mode | 243 | Unknown |
| 14 | Frontal_Parietal | 60 | Unknown | 106 | Unknown | 152 | Unknown | 198 | Default Mode | 244 | Unknown |
| 15 | Salience | 61 | Auditory | 107 | Unknown | 153 | Unknown | 199 | Unknown | 245 | Unknown |
| 16 | Ventral_Attention | 62 | Auditory | 108 | Unknown | 154 | Frontal_Parietal | 200 | Unknown | 246 | Unknown |
| 17 | Unknown | 63 | Default Mode | 109 | Unknown | 155 | Salience | 201 | Unknown | 247 | Unknown |
| 18 | Unknown | 64 | Default Mode | 110 | Unknown | 156 | Frontal_Parietal | 202 | Unknown | 248 | Unknown |
| 19 | Frontal_Parietal | 65 | Ventral_Attention | 111 | Unknown | 157 | Frontal_Parietal | 203 | Default Mode | 249 | Unknown |
| 20 | Salience | 66 | Visual | 112 | Unknown | 158 | Somato_Motor | 204 | Visual | 250 | Unknown |
| 21 | Frontal_Parietal | 67 | Visual | 113 | Unknown | 159 | Somato_Motor | 205 | Visual | 251 | Unknown |
| 22 | Frontal_Parietal | 68 | Visual | 114 | Unknown | 160 | Somato_Motor | 206 | Visual | 252 | Unknown |
| 23 | Somato_Motor | 69 | Frontal_Parietal | 115 | Unknown | 161 | Cingular_opercular | 207 | Visual | 253 | Unknown |
| 24 | Somato_Motor | 70 | Frontal_Parietal | 116 | Unknown | 162 | Cingular_opercular | 208 | Visual | 254 | Unknown |
| 25 | Somato_Motor | 71 | Unknown | 117 | Unknown | 163 | Auditory | 209 | Visual | 255 | Unknown |
| 26 | Somato_Motor | 72 | Visual | 118 | Unknown | 164 | Auditory | 210 | Visual | 256 | Unknown |
| 27 | Somato_Motor | 73 | Visual | 119 | Unknown | 165 | Frontal_Parietal | 211 | Visual | 257 | Salience |
| 28 | Cingular_opercular | 74 | Visual | 120 | Default Mode | 166 | Dorsal_attention | 212 | Visual | 258 | Subcortical |
| 29 | Cingular_opercular | 75 | Visual | 121 | Subcortical | 167 | Somato_Motor | 213 | Unknown | 259 | Subcortical |
| 30 | Frontal_Parietal | 76 | Visual | 122 | Subcortical | 168 | Cingular_opercular | 214 | Unknown | 260 | Subcortical |
| 31 | Frontal_Parietal | 77 | Visual | 123 | Subcortical | 169 | Cingular_opercular | 215 | Visual | 261 | Subcortical |
| 32 | Dorsal_attention | 78 | Visual | 124 | Subcortical | 170 | Subcortical | 216 | Visual | 262 | Subcortical |
| 33 | Somato_Motor | 79 | Visual | 125 | Subcortical | 171 | Somato_Motor | 217 | Auditory | 263 | Subcortical |
| 34 | Cingular_opercular | 80 | Visual | 126 | Subcortical | 172 | Somato_Motor | 218 | Somato_Motor | 264 | Subcortical |
| 35 | Cingular_opercular | 81 | Unknown | 127 | Subcortical | 173 | Auditory | 219 | Default Mode | 265 | Subcortical |
| 36 | Salience | 82 | Visual | 128 | Subcortical | 174 | Somato_Motor | 220 | Cingular_opercular | 266 | Unknown |
| 37 | Subcortical | 83 | Default Mode | 129 | Unknown | 175 | Somato_Motor | 221 | Cingular_opercular | 267 | Unknown |
| 38 | Somato_Motor | 84 | Somato_Motor | 130 | Unknown | 176 | Visual | 222 | Default Mode | 268 | Unknown |
| 39 | Somato_Motor | 85 | Default Mode | 131 | Unknown | 177 | Dorsal_attention | 223 | Unknown | | |
| 40 | Somato_Motor | 86 | Default Mode | 132 | Unknown | 178 | Unknown | 224 | Unknown | | |
| 41 | Somato_Motor | 87 | Visual | 133 | Unknown | 179 | Somato_Motor | 225 | Default Mode | | |
| 42 | Default Mode | 88 | Unknown | 134 | Default Mode | 180 | Salience | 226 | Default Mode | | |
| 43 | Frontal_Parietal | 89 | Somato_Motor | 135 | Unknown | 181 | Auditory | 227 | Default Mode | | |
| 44 | Dorsal_attention | 90 | Default Mode | 136 | Unknown | 182 | Default Mode | 228 | Unknown | | |
| 45 | Somato_Motor | 91 | Salience | 137 | Unknown | 183 | Default Mode | 229 | Default Mode | | |
| 46 | Auditory | 92 | Unknown | 138 | Default Mode | 184 | Frontal_Parietal | 230 | Default Mode | | |

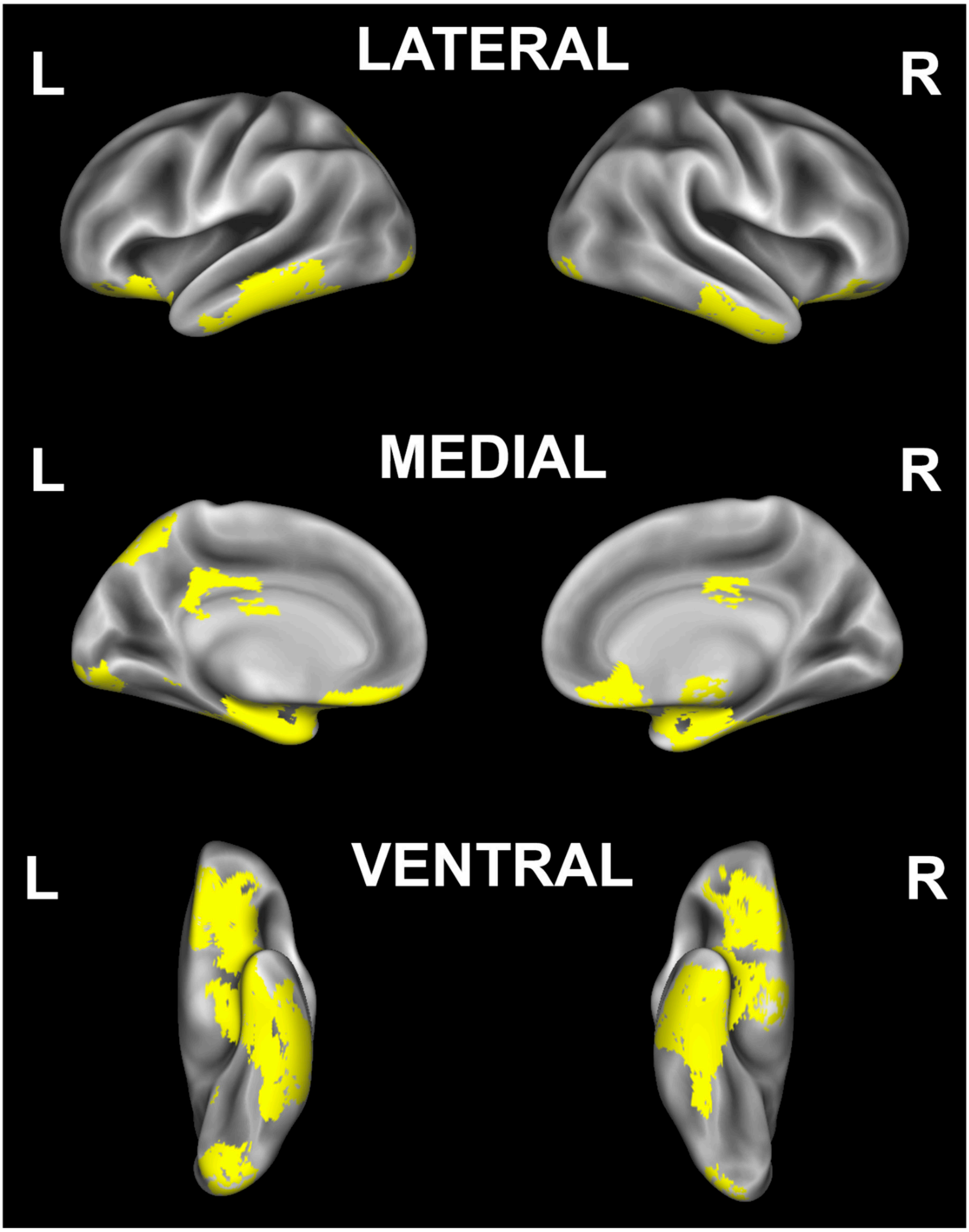


**Figure S1.** Visualization of unknown networks that was excluded in the present study. BioImage Suite Web Connectivity Viewer tool used to categorize unknown networks (BioImage Suite Web; https://bioimagesuiteweb.github.io/webapp/).

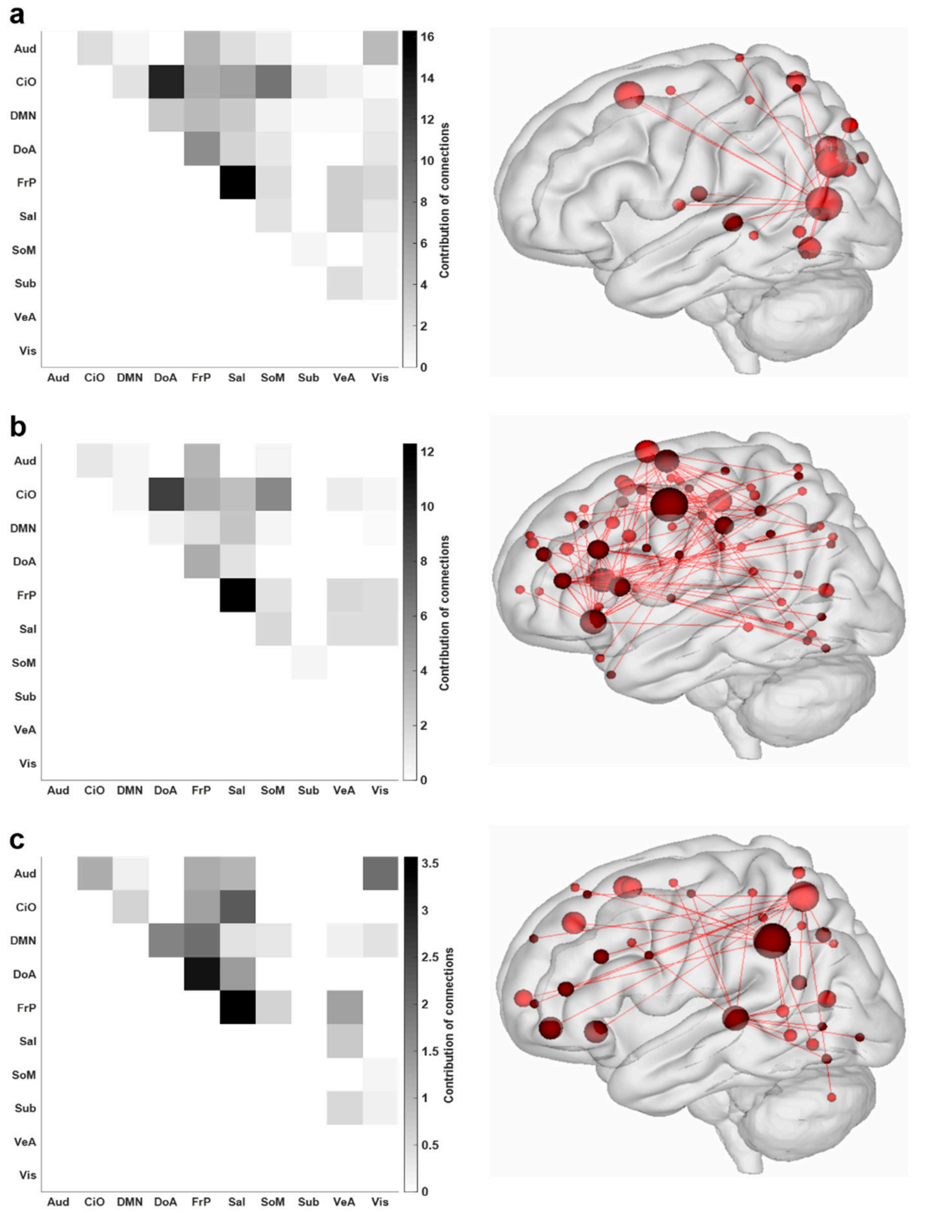


**Aud: auditory, CiO: cingular-opercular, DMN: default mode, DoA: dorsal attention, FrP: frontal parietal, Sal: salience, SoM: somato motor, Sub: subcortical, VeA: ventral attention, Vis: Visual**

**Figure S2.** FC differences between different conditions (a) FC differences between resting state and risk-rating task(b) FC differences between resting state and speed-rating task (c) FC differences between risk-rating task and speed-rating task. The color bars represent the portion of significantly differences FC between the conditions (%).

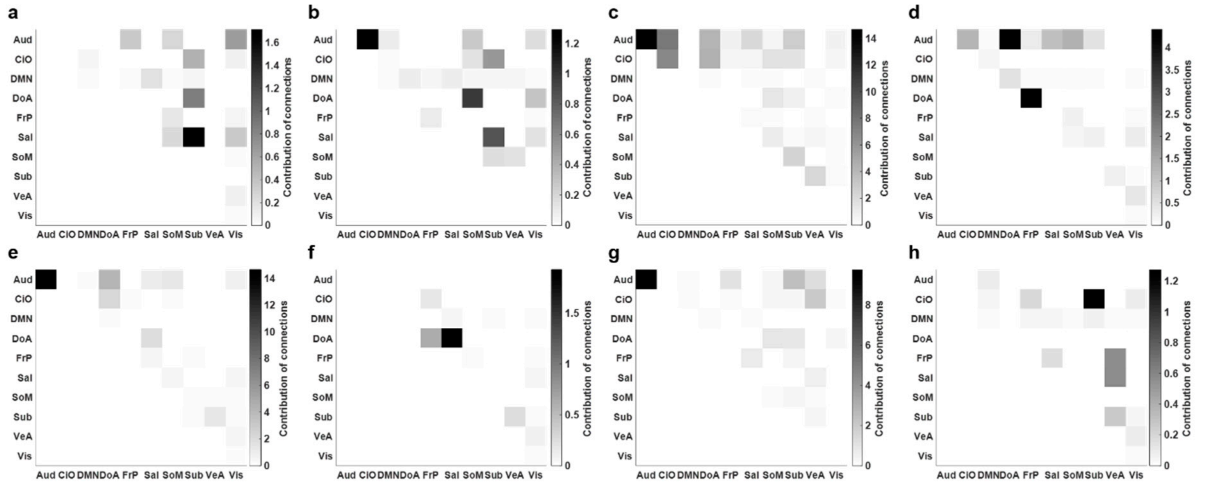

**Aud: auditory, CiO: cingular-opercular, DMN: default mode, DoA: dorsal attention, FrP: frontal parietal, Sal: salience, SoM: somato motor, Sub: subcortical, VeA: ventral attention, Vis: Visual**

**Figure S3.** Overall contribution of the connections for resting state functional connectivity to predict behavioral measures (a) positive network for predicting driving violations (b) positive network for predicting errors; (c) positive network for predicting lapses(d) positive network for predicting sensation seeking, (e) positive network for predicting impulsivity (f) negative network for predicting errors (g) negative network for predicting sensation seeking (h) negative network for predicting impulsivity. The color bars represent the contributed connection between the networks (%).

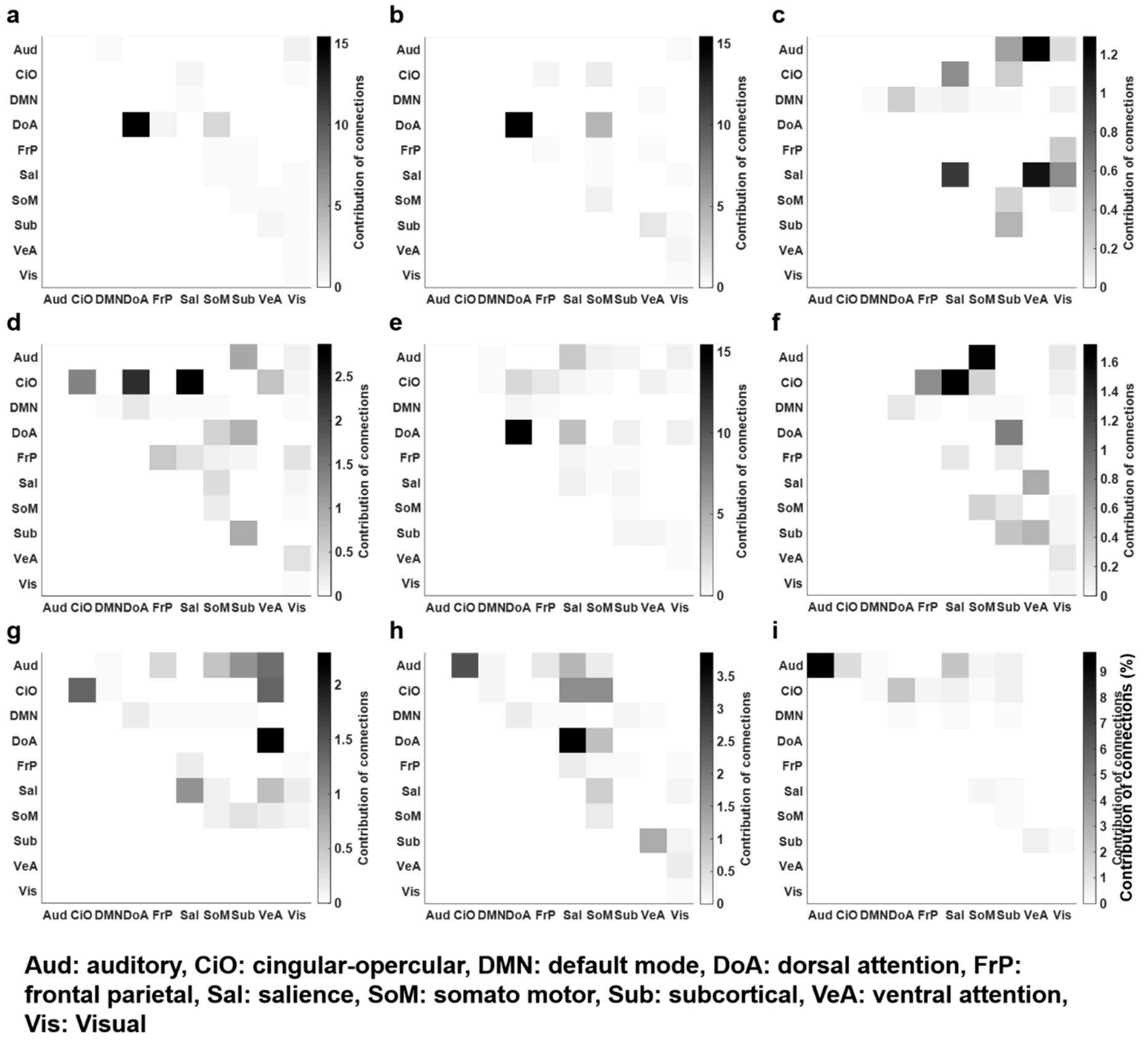


**Figure S4.** Overall contribution of the connections for task-based functional connectivity when assessing risk to predict behavioral measures. (a) positive network for predicting errors (b) positive network for predicting lapses (c) positive network for predicting sensation seeking (d) positive network for predicting impulsivity (e) negative network for predicting driving violations (f) negative network for predicting errors (g) negative network for predicting lapses (h) negative network for predicting sensation seeking (i) negative network for predicting impulsivity. The color bars represent the contributed connection between the networks (%).

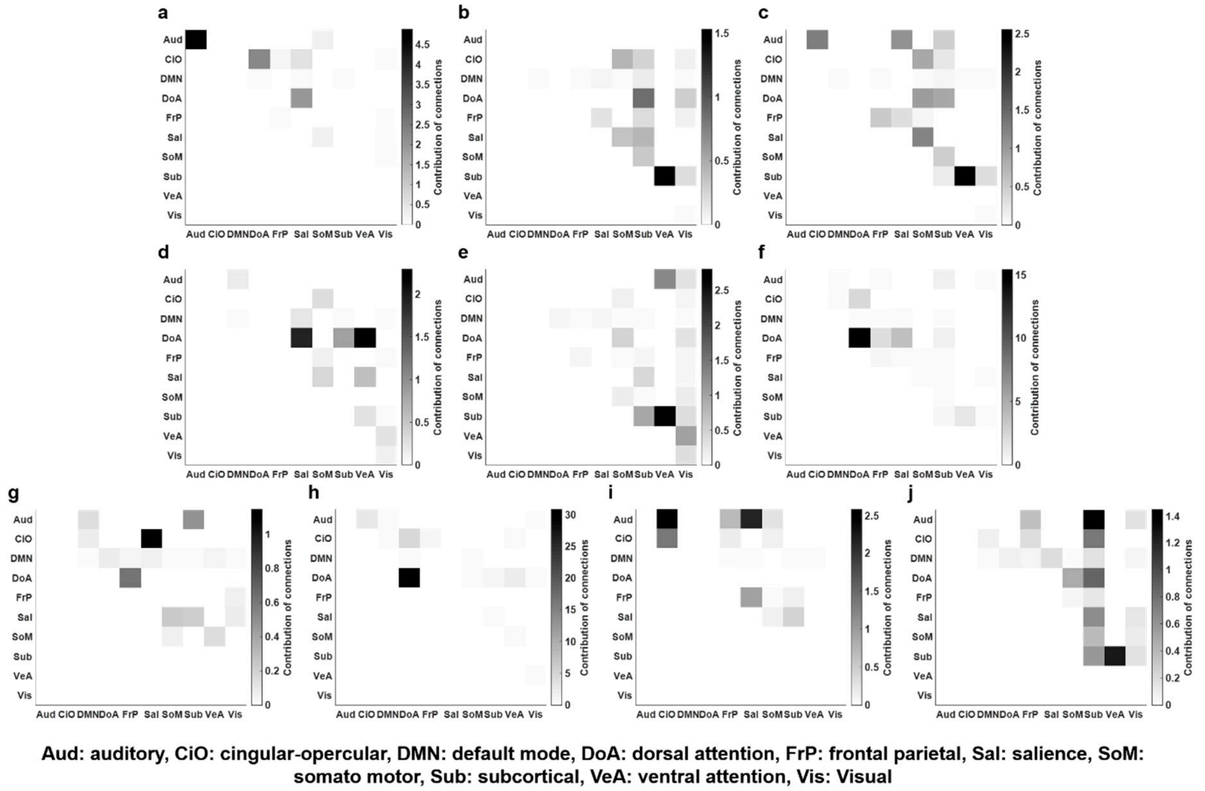


**Figure S5.** Overall contribution of the connections for task-based functional connectivity when assess speed to predict behavioral measures (a) positive network for predicting driving violations (b) positive network for predicting errors; (c) positive network for predicting lapses (d) positive network for predicting sensation seeking, (e) positive network for predicting impulsivity (f) negative network for predicting driving violations (g) negative network for predicting errors (h) negative network for predicting lapses (i) negative network for predicting sensation seeking (j) negative network for predicting impulsivity. The color bars represent the contributed connection between the networks (%).

## Supplementary methods

### Functional connectivity differences analysis

Before running functional connectome based behavioral prediction, first, FC differences between rsFC and tbFC were analyzed. For this, paired t-tests applied to the whole connectivity matrix between rsFC, risk-rating tbFC and speed-rating tbFC. Resulting p-values where Bonferroni corrected ($p < 0.05/31684$) for solving multiple comparison problems. Next, to visualize the number of significant networks connectivity differences between difference FC matrix, I calculated the number of significantly different connections between networks / total number of possible connections between the networks to investigate which connections between networks showed largest differences.

## Supplementary results

### Functional connectivity differences between conditions

Next, FC differences between rsFC, risk-rating tbFC and speed-rating tbFC was investigated. Results

showed that across all conditions, connectivity between frontal-parietal and salience networks showed the largest portion of significant differences (see Figure S2). Additionally, FC differences between rsFC with both risk rating tbFC and speed rating tbFC showed same high contributing networks from cingular-opercular with dorsal attention and somato-motor networks. Additionally, FC differences between risk-rating and speed-rating tbFC showed second highest portion between dorsal attention and frontal-parietal networks and third highest portion between cingular-opercular networks and salience networks. Overall, regions to make significant FC differences between resting state and task where similar regardless of task and frontal-parietal, salience, cingular_opercular and dorsal attention network showed common contribution to make significant FC differences between experimental conditions.
